# Forty Years of Inferential Methods in the Journals of the Society for Molecular Biology and Evolution

**DOI:** 10.1093/molbev/msad264

**Published:** 2024-01-03

**Authors:** Claudia A M Russo, Adam Eyre-Walker, Laura A Katz, Brandon S Gaut

**Affiliations:** Departamento de Genética, Universidade Federal do Rio de Janeiro, Rio de Janeiro, Brazil; School of Life Sciences, University of Sussex, Brighton, UK; Department of Biological Sciences, Smith College, Northampton, MA, USA; School of Biological Sciences, University of California, Irvine, CA, USA

**Keywords:** Society for Molecular Biology and Evolution, anniversary celebration, inferential methods, software packages, SMBE journals

## Abstract

We are launching a series to celebrate the 40th anniversary of the first issue of *Molecular Biology and Evolution*. In 2024, we will publish virtual issues containing selected papers published in the Society for Molecular Biology and Evolution journals, *Molecular Biology and Evolution* and *Genome Biology and Evolution*. Each virtual issue will be accompanied by a perspective that highlights the historic and contemporary contributions of our journals to a specific topic in molecular evolution. This perspective, the first in the series, presents an account of the broad array of methods that have been published in the Society for Molecular Biology and Evolution journals, including methods to infer phylogenies, to test hypotheses in a phylogenetic framework, and to infer population genetic processes. We also mention many of the software implementations that make methods tractable for empiricists. In short, the Society for Molecular Biology and Evolution community has much to celebrate after four decades of publishing high-quality science including numerous important inferential methods.

## Forty Years of the Society for Molecular Biology and Evolution Journals

*Molecular Biology and Evolution* (*MBE*) has reached its fortieth anniversary. The journal, and ultimately the Society for Molecular Biology and Evolution (SMBE), traces its origin to a June 1982 meeting held at the State University of New York at Stony Brook. Following a symposium on the “Evolution of Genes and Proteins,” the participants gathered to discuss an idea for a new journal. They saw a need for a journal that bridged the gap between molecular and evolutionary biology, provided a high-quality forum for publications at an accessible cost, and was governed by the scientific community. The first issue of *MBE* was published only 18 months later, in December 1983.

**Fig. 1.**
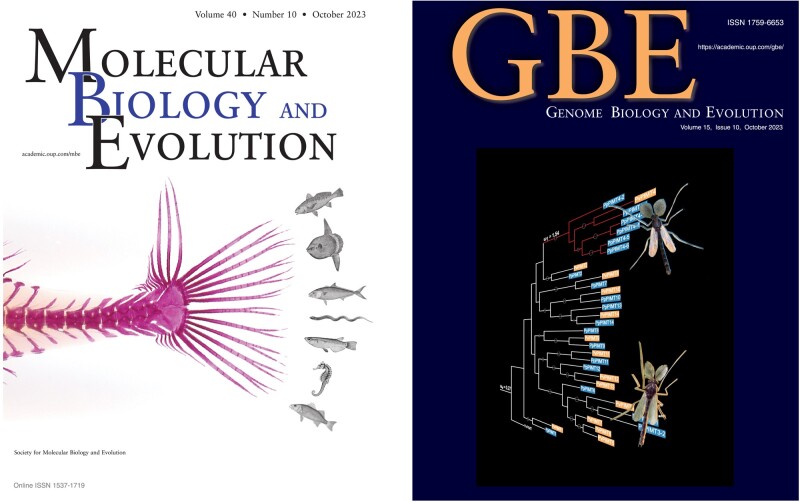
Recent covers from the two SMBE journals, MBE (left) and GBE (right).

The success of *MBE* contributed to the formation of the SMBE in 1992. SMBE has grown as a society and supported the founding of a second journal, *Genome Biology and Evolution* (*GBE*), in 2009 (Fig. 1). Originally designed to focus on emerging genome-scale data, *GBE* was among the first society-owned open-access journals. Recently, *MBE*, following *GBE*'s lead, has also become open access. In keeping with the goals of the founding group, the cost of publishing in the SMBE journals remains modest, and both journals have an inclusive waiver policy with a commitment to publish high-quality science regardless of an author’s ability to pay publication costs. The proceeds from SMBE publishing support other SMBE activities, including the annual meeting, IDEA initiatives, satellite meetings, and various awards and fellowships.

To mark our 40-year anniversary, the SMBE journals are launching a collaborative year-long celebration. Throughout 2024, *MBE* and *GBE* will publish perspectives centered on topics of both contemporary and historical significance within our field. Each perspective will be complemented by virtual issues housing selected publications from both journals that highlight work relevant to the monthly topic. In addition to their availability on journal websites, the virtual issues can be accessed from the newly launched website that represents the SMBE family of journals (www.academic.oup.com/smbejournals). We hope that the perspectives and virtual issues will encourage our community to celebrate the lasting impacts of journals built by, and for, the community.

**Fig. 2.**
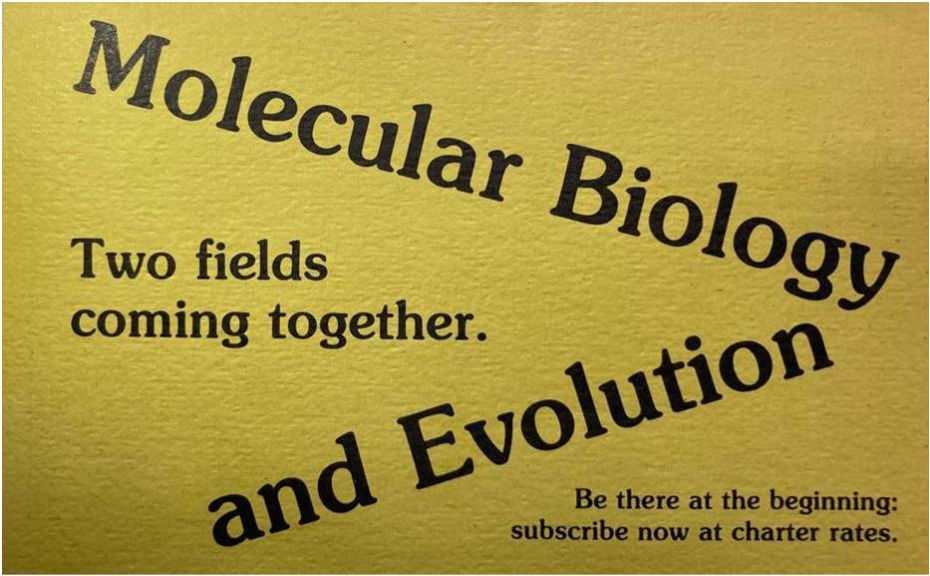
The first issue of *MBE* included a flier that advertised the intention to merge the fields of molecular biology with evolutionary biology.

The monthly topics in the anniversary series will be as varied as the papers in the journals, including topics like testing for selection, human diversification, the mechanisms and consequences of recombination, sex chromosomes, and microbial diversity. For this first installment, we have created virtual issues and this perspective to highlight papers on inferential methods in evolutionary biology. Over the last four decades, our journals have played a central role in developing methods for the study of molecular evolution, resulting in a rich literature of highly cited studies. In fact, the literature is so vast that we cannot hope to mention all the impactful papers here. Instead, we focus on a few key research areas (e.g. phylogenetic inference, hypothesis testing in a phylogenetic framework, and population genetic analysis). We hope that this perspective highlights the variety of methodological improvements that have been published in the SMBE journals and illustrates the success of the founders’ goal to bridge gaps between molecular and evolutionary biology (Fig. 2).

## An Overview of Methods Published in SMBE Journals

### Phylogenetic Inference

The SMBE journals have played a pivotal role in the development and application of molecular phylogenetic methods. One example is the neighbor-joining algorithm (Saitou and Nei 1987), which remains the most cited single publication in the SMBE journals with more than 50,000 citations. Neighbor-joining has had a lasting influence because it is easily implemented, accurate if distances are unbiased (Rzhetsky and Nei 1993), and lightning fast, even for very large numbers of sequences (Huelsenbeck 1995).

Other tree-building methods presented in our journals include BIONJ (Gascuel 1997), FastTree (Price et al. 2009), minimum-evolution (Rzhetsky and Nei 1992), weighted neighbor-joining (Bruno et al. 2000), generalized neighbor-joining (Pearson et al. 1999), NJML (Ota and Li 2000), and quartet puzzling (Strimmer and von Haeseler 1996). An alternative to these bifurcating tree-building methods is family-joining, which is for taxa that have been sampled over evolutionary time when a strictly bifurcating tree may not be appropriate (Kalaghatgi et al. 2016). Family-joining is a distance method in which operational taxonomic units (OTUs), such as viruses, can be placed not only at external branches but also at internal vertices. Another set of papers have focused on network construction, which can be more relevant for building intraspecific trees with low genetic distances among OTUs. Network approaches published in our journals include a combinatorial method (Huson and Scornavacca 2011), median-joining (Bandelt et al. 1999), neighbor-net (Bryant and Moulton 2004), and the Bayesian inference for species’ network (Zhang et al. 2018).

SMBE journals have played a leading role in adopting Bayesian approaches for constructing phylogenies. For example, the work of Yang and Rannala (1997) was among the first to implement the Markov chain Monte Carlo (MCMC) algorithms for Bayesian phylogenetics. This was a significant step forward and merits recognition, because MCMC has become the go-to approach to approximate the posterior distribution of evolutionary parameters.

Many tree-building methods use a concatenated (multigene) alignment to build a single phylogenetic tree. For the same set of OTUs, however, individual genes may yield different topologies due to distinct evolutionary histories such as gene duplications/loss, lateral gene transfers, or the presence of genetic polymorphism in the ancestral species. Pamilo and Nei (1988) provided the theoretical foundation to phylogenetic reconstructions based on multispecies coalescence models. Their method provides analytical solutions for the probability of topological matches between gene and species trees. In this approach, a phylogenetic tree is first built for each available marker, and then individual gene trees are summarized into a single species tree, analyzing conflicts between gene trees. Similar methods have been published across our journals, including extensions of coalescent approaches (Ané et al. 2007; Heled and Drummond 2010) and methods suitable to large data sets (Ullah et al. 2015).

A newer method for testing incongruences between gene trees is PhylteR, which is useful for phylogenomic data sets. In this case, distance matrices built from each marker are compared in order to detect (and possibly remove) outlier sequences that are the OTUs that do not follow the general pattern (Compte et al. 2023). In 2012, Bryant et al. introduced a new method, for biallelic and nonlinked markers, to build a species tree considering all possible gene trees (Bryant et al. 2012). In this method, rather than integrating over all possible genealogies to determine the probability of data given the species tree, the authors developed a pruning algorithm for analytical calculation of this value, bypassing the construction of individual gene trees.

It might be useful to delimit species using molecular markers, particularly if the taxonomic group presents few conspicuous morphological characteristics. Using the multispecies coalescent model in a Bayesian framework, Yang and Rannala (2014) developed a method to infer species delimitation and to estimate the species phylogeny at the same time. This new method differed from previous algorithms that required a guiding phylogeny.

Studies published in our journals have also advanced our ability to evaluate the robustness of phylogenetic tree clades for large data sets, where algorithm speed is critical. These methods include UFBoot, an ultrafast version of the bootstrap test of branch support (Minh et al. 2013; Hoang et al. 2018) and a coalescent-based method that quickly evaluates branch support from quartet frequencies (Sayyari and Mirarab 2016). Earlier studies have provided important insights about reliability estimates; for example, Hedges (1992) estimated the number of bootstrap replications needed for a precise estimate of the bootstrap support value.

Phylogenies cannot be produced without quality alignments, and the SMBE journals have contributed to this important step, publishing versions of the popular MAFFT (Katoh and Standley 2013) and GBlocks (Castresana 2000) software. MAFFT allows rapid generation of multisequence alignments under a variety of parameterizations, while GBlocks evaluates alignment quality, identifies the most conservative (and presumably trustworthy) blocks of alignment, and allows the user to discard more variable sections with poorer alignments.

Most methods of phylogenetic inference require nucleotide substitution models. But how accurate and general are these models, and which one best suits specific data sets? The availability of numerous nucleotide substitution models creates the challenge of model choice because the wrong model can affect both phylogenetic inference and other statistical analyses. Our journals have led the field in proposing and evaluating nucleotide substitution models, ranging from the first model published in *MBE* (Tajima and Nei 1984) to refinements that improve their generality (Tamura and Nei 1993). The journals have also published several methods to test the fit of evolutionary models, such as PartitionFinder (Lanfear et al. 2012; Lanfear et al. 2017), SMS (Lefort et al. 2017), ModelTest-NG (Darriba et al. 2020), jModelTest (Posada 2008), and additional tests that take codon position into account (Shapiro et al. 2006). Huelsenbeck et al. (2004) expanded the pool of models for comparison using Bayes factors and a reversible jump MCMC; their approach included the possibility to test among nonnested models (Huelsenbeck et al. 2004). Using empirical data, it is a complex task to ascertain the correct model and it is useful to know if the tree-building method is robust against model violations. To assess the scale and impact of model violations for phylogenetic inference, Naser-Khdour et al. (2019) implemented a maximal matched-pairs test of homogeneity.

Finally, rooting an evolutionary tree is a critical step in phylogenetic inference, as only rooted trees can be directly related to divergence times. Lake et al. (2007) developed the top-down indel rooting method that uses nonubiquitous genes to root the tree of life. Unlike previous methods, this rooting method uses information not only on indel gains and losses but also on gene gains and losses (Lake et al. 2007).

### Hypothesis Testing in a Phylogenetic Framework

Phylogenies allow us to address questions about species relationships, but they also form the basis for downstream analyses. Some fundamental analyses use phylogenies to visualize data (phenotypic, experimental, clinical, etc.) onto a phylogenetic framework. For cases like these, Yu et al. (2018) developed an R package named ggtree to map and visualize data.

Other applications require additional inference. One useful goal is to produce a timetree that places divergence times on interior nodes. The construction of timetrees has a lengthy history in the SMBE journals. For example, Takezaki et al. (1995) developed a method based on the assumption of a strict molecular clock. Their application used a single calibration time point and allowed users to remove lineages that deviated significantly from the rate constancy assumption. This linearized tree method differs from the current relaxed-clock methods that are currently popular. For example, Yang and Rannala (2005) developed a Bayesian MCMC algorithm that allows for multiple and softbound calibration priors for estimating divergence times, contrasting with the single or hardbound time priors of earlier methods. New methods can even accommodate phylogenetic uncertainty (Baele et al. 2012), and most of them are able to estimate divergence times when evolutionary rates vary across lineages (Sanderson 1997; Rambaut and Brooman 1998; Thorne et al. 1998; Sanderson 2002).

Many of these methods employ Bayesian approaches, which can be computationally intensive and prohibitive with large data sets. RelTime is a faster algorithm for estimating timetrees. Although originally published elsewhere (Tamura et al. 2012), recent advances and evaluations of RelTime have been published in *MBE*, including the theoretical foundation of the method (Tamura et al. 2018) and evaluations based on simulated (Filipski et al. 2014) and empirical (Mello et al. 2017) data sets.

We have also published a helpful online resource called TimeTree of Life (Kumar et al. 2017). This project assembles information on the timescale of life, in a similar way to the Tree of Life project (Maddison et al. 2007), but focuses on divergence times rather than clades. This resource includes searchable divergence time data from ∼4,000 studies across ∼148,000 species and is now in its fifth release (Kumar et al. 2022). Apart from time estimates, TimeTree also allows users to explore timetrees that are extracted, for a given taxonomic group, from the global timetree.

Another major theme, which is closely related to estimating and testing molecular clocks, is estimating rates of nonsynonymous (*d*_N_) and synonymous (*d*_S_) evolution and their ratio (*d*_N_*/d*_S_) along evolutionary branches of a tree. The use of *d*_N_*/d*_S_ as a signal of adaptive evolution was fueled in part by advances like the publication of a simple distance method that estimates *d*_N_ and *d*_S_ between pairs of OTUs (Nei and Gojobori 1986). The later publication of codon-based models of nucleotide substitution (Goldman and Yang 1994; Muse and Gaut 1994) laid the groundwork for maximum likelihood hypothesis testing of *d*_N_*/d*_S_ on evolutionary trees, including the capability to test for adaptive evolution on individual branches and on specific codons (Yang and Nielsen 2000, 2002).

### Population Genetic Analysis

Population genetics has been a prominent topic in SMBE journals since their inception. In fact, the first issue featured a paper by Motoo Kimura, which extended his “neutral theory of molecular evolution and polymorphism” to estimate the fraction of selectively neutral alleles among new mutations from what became known as the site frequency spectrum (Kimura 1983). This method presaged those that estimate the distribution of fitness effects (DFE) and the rate of adaptive evolution (Eyre-Walker and Keightley 2009; Huang et al. 2021). These and similar methods have spawned numerous empirical studies (e.g. Slotte et al. 2010; Gossmann et al. 2012; Tsagkogeorga et al. 2012; Campos et al. 2018) that have provided insights into both the evolutionary processes and the variation of DFEs across populations and species.

Another major theme has been analyzing the effect of evolutionary forces, such as gene flow and drift, on rates of population divergence. Before these forces can be characterized, however, it may be important to first identify separate populations. To this end, Hudson et al. (1992) published a widely used test for detecting geographic subdivision among populations. Once populations are identified, one can model the process of divergence in the face of gene flow between populations. Also, Hey (2010) contributed seminal work to this topic by extending isolation–migration models to multiple populations, which also provided a means to estimate divergence times and migration rates across populations. Related innovations include the ABBA–BABA statistics (Durand et al. 2011) and their extensions (Martin and Amos 2021). These statistics can detect the signatures of introgression between populations and, in some cases, can also infer the direction of historical introgression events (Martin and Amos 2021).

Population divergence is affected by genetic drift, which is a function of demographic history, another recurring focus of our journals. The inference of demography has undergone a revolution with the introduction of methods that estimate the chronological history of effective population size (*N*_e_) without a predefined demographic model. These methods rely on the temporal rate of coalescence in a genealogy, and they typically produce a plot of estimated *N*_e_ over time—i.e. a “skyline plot.” Several refinements and improvements to this approach have been published in SMBE journals, including generalizing skyline plots for cases with low divergence (Strimmer and Pybus 2001), estimating plots directly from sequence data rather than from an inferred genealogy (Drummond et al. 2005), and improving temporal smoothing and inference (Minin et al. 2008).

We have touched on many themes in population genetics, but some might argue that *the* major theme is detecting adaptive evolution. This topic is so broad, with such a rich history in our journals, that one of the perspectives in the 40th anniversary series will focus on detecting deviations from neutrality. In this context, we want to draw attention to three basic points. First, the historical effects of demographic changes often complicate the inference of selection, because these processes can produce similar diversity patterns (Johri et al. 2022). Substantive efforts have been invested into controlling for demographic history prior to inferring selection using both empirical (Tenaillon et al. 2004; Stajich and Hahn 2005) and modeling approaches. An example of the latter is *dadi*, which uses allele frequency information to infer demographic history (Gutenkunst et al. 2009) but can incorporate selection on single sites and predict the joint distribution of selected alleles among populations. An update in *MBE* improves the performance of *dadi* and applies it to more than three populations (Gutenkunst 2021).

Second, our community has shown strong interests in detecting and understanding the behavior of selective sweeps. For example, SweeD adapted the CLR (Composite Likelihood Ratio) test (Nielsen et al. 2005) to a high-performance computing environment (Pavlidis et al. 2013). The CLR test is most effective at detecting hard sweeps caused by a single, new adaptive mutation. However, both theory (Pennings and Hermisson 2006) and empirical data (Schrider and Kern 2017) suggest that soft sweeps—i.e. sweeps that result from multiple (often competing) alleles—are likely to be common in nature. Haplotype-based methods have been particularly helpful for detecting soft sweeps. Excoffier and Slatkin (1995) produced key early work in this area by presenting methods to estimate haplotype frequencies; Ferrer-Admetlla et al. (2014) introduced a summary statistic based on haplotype frequencies to test for selection, with improvements in statistical power compared to previous methods; and Harris and DeGiorgio (2020) published an approach that is useful for detecting both hard and soft sweeps from the haplotype frequency spectrum. More recent publications include a haplotype-based method (Flex-sweep) that utilizes convolutional neural networks (Lauterbur et al. 2023) and an efficient haplotype-based approach suitable for large data sets (Kirsch-Gerweck et al. 2023).

Many methods for detecting both soft and hard sweeps focus on single loci or genomic regions, but adaptation is often polygenic (Pritchard et al. 2010). The detection of polygenic adaptation requires different approaches, and one valuable approach for detecting potential polygenic adaptation was published in *MBE*. Frichot et al. (2013) applied a “latent factor mixed model” (LFMM) to identify alleles (i) with frequency distributions that are not easily explained by population structure and (ii) that are associated with other variables, such as bioclimatic measures. Alleles that fit both of these criteria are candidates for contributing to local adaptation that may be polygenic. A subsequent publication updated the LFMM approach (Caye et al. 2019).

Finally, the prevalence and role of balancing selection in the evolutionary process remains poorly understood. However, many of the methods that have been used to detect balancing selection have been published in SMBE journals (Hunter-Zinck and Clark 2015; Siewert and Voight 2017; Bitarello et al. 2018; Cheng and DiGiorgio 2019).

### Software Packages

In addition to publishing methods, the SMBE journals have published numerous popular software packages that make methods accessible to our community. Many of these packages embed features like alignment, model choice, phylogenetic inference, and associated downstream analyses. MEGA is the most prominent of these packages. Although MEGA was first announced elsewhere (Kumar et al. 1994), several versions have been published in *MBE* (e.g. Tamura et al. 2007; Kumar et al. 2018; Tamura et al. 2021). These more recent versions have widely increased its scope, including more statistical analyses and various tree-building and timetree methods. Taken together, the collection of MEGA papers has been cited more than any other set of publications in our journals, which is a strong testament to both their scientific contributions and their practical importance. The fast RelTime analyses have been implemented in MEGA (Tamura et al. 2021), allowing users to evaluate the robustness of time estimates by, for example, comparing divergence times using distinct calibration sets. Three protocol papers, which detail pipelines for empirical analyses, have been published that focus on analyses using MEGA. These protocols guide MEGA users for building phylogenetic trees (Hall 2013), performing bootstrap tests of branch support (Russo and Selvatti 2018), and estimating divergence times (Mello 2018).

Our journals have published other prominent software packages like DAMBE (Xia 2013), PAML (Yang 2007), IQ-TREE (Nguyen et al. 2015; Minh et al. 2020), and SEAVIEW (Gouy et al. 2010). DAMBE covers most of the major steps of phylogenetic inference, such as sequence alignment, model selection, and tree building, as well as analyses like codon bias detection and inferring the isoelectric point of a particular enzyme in a solution. SEAVIEW is useful for aligning sequences, to inspect and edit alignments, and to concatenate individual blocks of alignment. It also performs some phylogenetic inference, although it is not its major focus. PAML features a large number of evolutionary models that can be used to compare and test phylogenetic trees and to test alternative biological hypotheses. Importantly, PAML can also be used to reconstruct ancestral gene and protein sequences. Many nonsynonymous (*d*_N_) and synonymous (*d*_S_) evolution and similar methods are also available in PAML (Yang 2007) and HYPHY (Pond et al. 2005); these programs are largely responsible for the burst of interest in investigating adaptive molecular evolution in coding sequences during the 2000s. *MBE* has published recent updates to both programs (Xu and Yang 2013; Pond et al. 2020) as well as FUBAR, an approach that can rapidly detect positive and purifying natural selection with large data sets (Murrel et al. 2013). A useful PAML protocol to estimate synonymous and nonsynonymous distances and to detect positive selection was recently published in *MBE* (Álvarez-Carretero et al. 2023).

The IQ-TREE package is another popular and user-friendly program. It includes a search algorithm that greatly improves exploration of tree space, yielding ML trees with higher likelihoods (Nguyen et al. 2015). In the latest release, search algorithms are able to use multicore CPUs and a parallel MPI (Message Passing Interface) system to speed analyses (Minh et al. 2020). The latest version incorporates over 200 time-reversible evolutionary models for DNA, protein, codon, binary, and multistate morphological data, as well as the ultrafast bootstrap and ModelFinder algorithms.

For timetree analysis, a popular program is BEAST, which can use either strict- or relaxed-clock models in a Bayesian framework. The species tree inference using biallelic markers, such as SNPs and AFLPs, was also implemented in BEAST (Bryant et al. 2012). BEAST has now been updated elsewhere (Suchard et al. 2018), but an earlier version was published in SMBE journals (Drummond et al. 2012). We have also published BEAST-related protocols for estimation of past population dynamics (Hill and Beale 2019) and for phylogeographic inference (Dellicour et al. 2021).

The latest update of the BUSCO method (Simão et al. 2015) was published in *MBE* (Manni et al. 2021). The primary purpose of BUSCO is to provide quality control on new genome assemblies by assessing the complement of near-universal single-copy orthologs within an assembly. BUSCO works with taxon-specific databases of single-copy orthologs, and the latest versions include greatly expanded representation of eukaryotic, prokaryotic, and viral genes. The new version of BUSCO also enables automatic database selection based on the phylogenetic insertion of input sequences.

With respect to population genetic analyses, the SMBE journals have published packages such as DNASP (Rozas et al. 2017), POPGENOME (Pfeifer et al. 2014), FUBAR (Murrell et al. 2013), and SLIM3 (Haller and Messer 2019a). The latest version of DNASP is configured for large data sets and is particularly suitable for genomic partitioning data such as RADseq. The POPGENOME package uses the R environment to process genome-scale data; it offers a range of population genetic analyses, including neutrality tests, population differentiation analysis, and recombination and disequilibrium detection tests. Finally, the SLiM forward simulation software is a popular and invaluable tool for population genetic analyses; *MBE* has published updates to this software (Haller and Messer 2017, 2019a) and a step-by-step protocol for new users (Haller and Messer 2019b).

It is important to emphasize that these software packages do not simply provide access to various methods and algorithms; new versions often introduce new and more accurate methods, enabling users to pursue their own analytical designs. Furthermore, the packages are freely available, usually multiplatform, and often feature detailed manuals with user-friendly graphical interfaces. In many cases, they can run on regular desktop computers without large memory or disk space requirements. These factors are aligned with SMBE journals’ policies and traditions and could explain their enormous success and critical role in bringing new members to our molecular evolution community.

## The Next 40 Years

The SMBE journals are thriving, publishing over 500 articles a year that merge the approaches of molecular biology, computational biology, statistics, genomics, and evolutionary theory. They have grown to encompass new fields and technologies associated with functional and evolutionary genomics, and they continue to be ranked among top evolutionary biology and genetics journals. Moreover, the SMBE journals remain dedicated to ensuring that data and tools are fully accessible from the point of publication onward, exemplified by *GBE*'s initiative to manually verify data availability statements in all accepted manuscripts.

This perspective, along with the accompanying virtual issue, should convince readers that authors publishing in SMBE journals have had a major impact on the methods and software used for evolutionary inference. Given the success of the last 40 years, we cannot help but wonder what the next 40 will bring. Although we are well into the “postgenomics era,” the pace of methodological advances for interpreting genomic data has not slowed. Some of these advances are fueled by the relative ease of procuring new data, contributing to ever larger data sets. Other innovations are fueled by new data types—e.g. single cell expression, chromatin and epigenomic data, improved biochemical structures, 3D genome topologies, and long-read assemblies.

As data sets expand, computational and statistical approaches will continue to evolve. Machine learning methods are increasingly applied to numerous problems in our field, including phylogenetic inference (Azouri et al. 2021), coevolutionary rates of branches on a phylogeny (Tamura et al. 2021), model selection for phylogenetic inference (Abadi et al. 2020), detection of selective sweeps (Lauterbur et al. 2023), and other population genetic inferences (Flagel et al. 2019). Likewise, the broader field of artificial intelligence (AI) is already having substantive impacts, both formally and informally. Researchers are, for example, using ChatGPT and other AI platforms for editing, coding, and preliminary data analyses. The application of these methods across distributed computing platforms will yield a new generation of methods for the curation, analysis, and interpretation of large data sets. We want to be at the forefront of these developments over the next four decades, just as our journals have contributed to revolutions in genomic, genetic, and structural analyses over the last four.

**Fig. 3.**
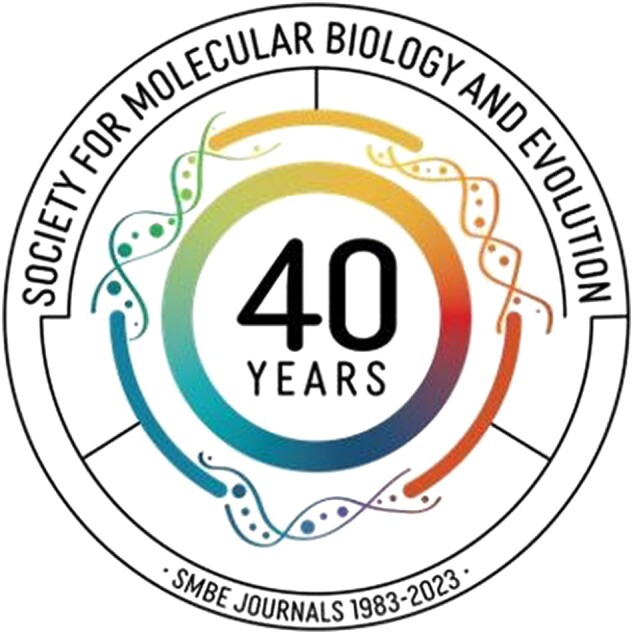
A logo to celebrate the 40th anniversary, which was designed by SMBE member Ana Carolina Martins Junqueira from the Federal University of Rio de Janeiro, Brazil.

We conclude this perspective by inviting you to celebrate the 40th anniversary (Fig. 3) by perusing the upcoming virtual issues and accompanying perspectives. But you need no invitation, because the SMBE journals are your journals. They were established by our burgeoning community in 1983, and they continue to be managed by the community and for the benefit of the community. They rely on your expert opinions as authors, reviewers, and editors. If you have authored, reviewed, edited, or read papers in *GBE* and *MBE*, then this is your celebration!
